# Supplementary material for: A Biological and Immunological Characterization of Schistosoma Japonicum Heat Shock Proteins 40 and 90α
Source: Int J Mol Sci. 2020 Jun 4;21(11):4034. doi: 10.3390/ijms21114034 (PMC7312537; doi:10.3390/ijms21114034)
Supplement: Supplementary file 1 [file ijms-21-04034-s001.zip › ijms-787217-final supplementary/Fig Sup..pdf]

Fig. S1. Sjp40

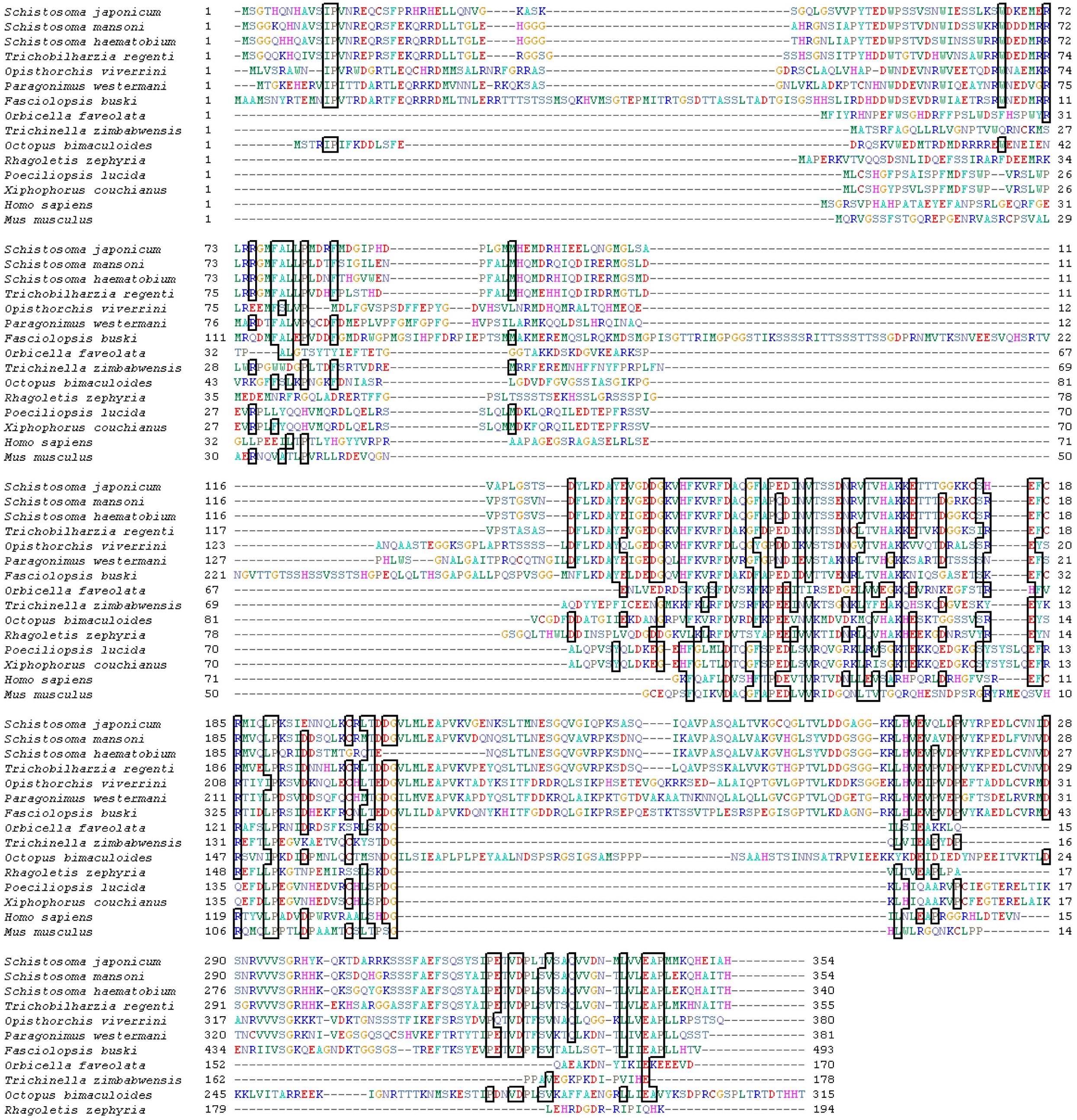

Fig. S2. Sjp90

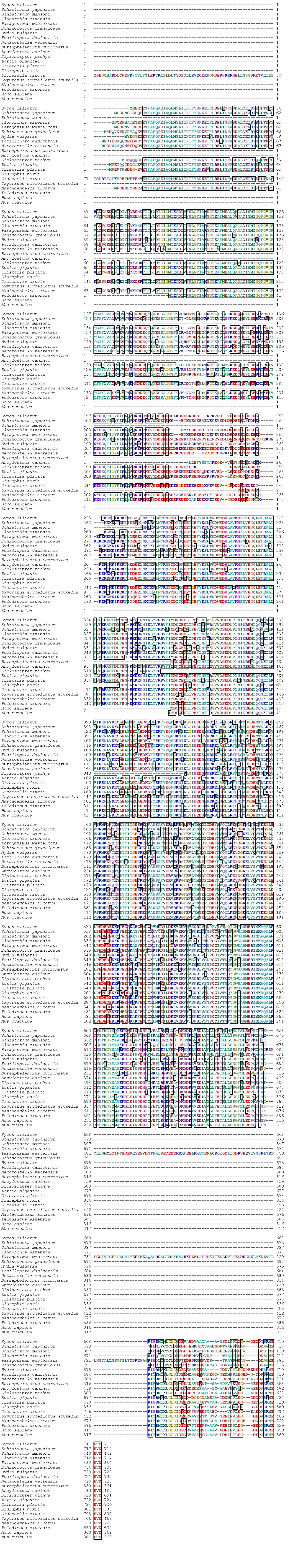

Fig. S3

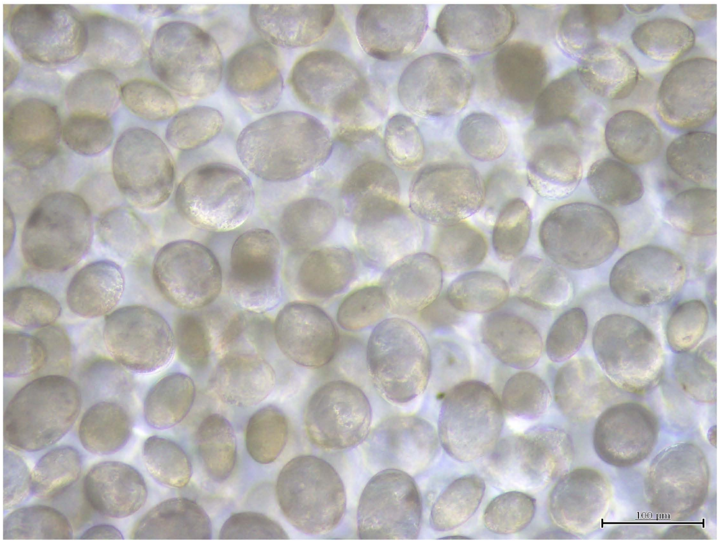

Fig. S4

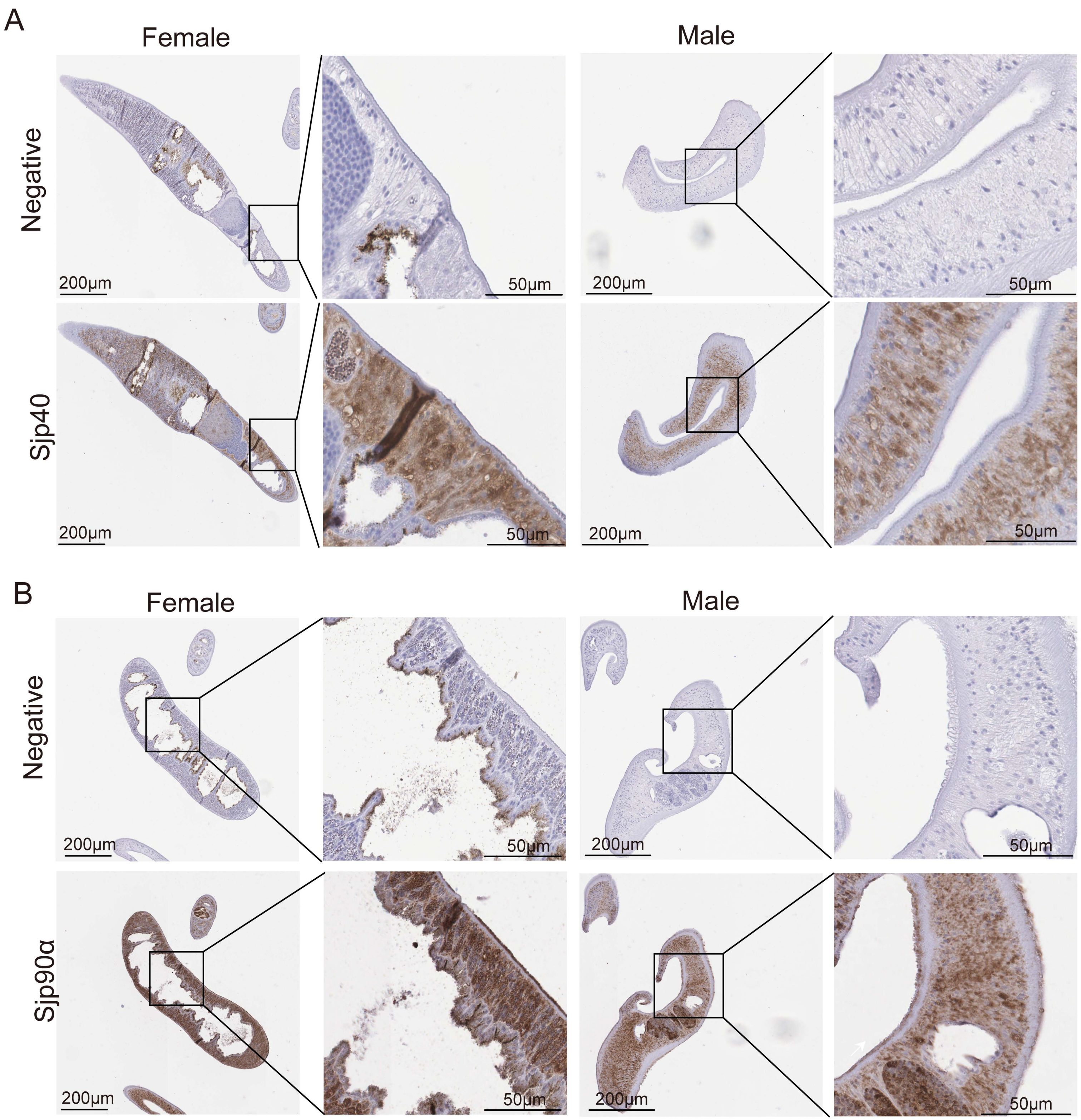
A
Female
Male
Negative
Sjp40
200μm
50μm
200μm
50μm
200μm
50μm
200μm
50μm
B
Female
Male
Negative
Sjp90α
200μm
50μm
200μm
50μm
200μm
50μm
200μm
50μm
