# Supplementary material for: A Biological and Immunological Characterization of Schistosoma Japonicum Heat Shock Proteins 40 and 90α
Source: Int J Mol Sci. 2020 Jun 4;21(11):4034. doi: 10.3390/ijms21114034 (PMC7312537; doi:10.3390/ijms21114034)
Supplement: Supplementary file 1 [file ijms-21-04034-s001.zip › ijms-787217-final supplementary/ijms-787217-supplementary table.docx]

| **Supplementary S1 Table. Details of the primers used in the real time PCR** | | |
| --- | --- | --- |
| Source | Gene | Sequence (5'- 3') |
| *Schistosoma japonicum* | *Sjp40* | F: CCCACATGATCCGTTGGGAA |
|  |  | R: CCATCATCGCCGACTTCGTA |
|  | *Sjp90a* | F: ACGTTTCTCGTATGAAGCCTGA |
|  |  | R: TTCAAAGCCGCGCTGAGTAA |
|  | *PSMD4* | F: CGTTCAGCTCCTGGAAATCTACTAGTT |
|  |  | R: CACGCCTAAACAATGCTGACG |
| Human | *α-SMA* | F: CAGGGCTGTTTTCCCATCCAT |
|  |  | R: GCCATGTTCTATCGGGTACTTC |
|  | *Col1a1* | F: GTCGAGGGCCAAGACGAAG |
|  |  | R: CAGATCACGTCATCGCACAAC |
|  | *β-actin* | F: CATGTACGTTGCTATCCAGGC |
|  |  | R: CTCCTTAATGTCACGCACGAT |
